# Left atrial function after myocardial infarction in swine

**DOI:** 10.1186/1532-429X-17-S1-P142

**Published:** 2015-02-03

**Authors:** Dana C Peters, Edgar J Diaz, Alda Bregazi, Stephanie L Thorn, Mitchel R Stacy, Christi Hawley, Albert J Sinusas

**Affiliations:** Diagnostic Radiology, Yale School of Medicine, New Haven, CT USA; Internal Medicine, Yale School of Medicine, New Haven, CT USA

## Background

Atrial fibrillation is the most common arrhythmia in United States, and is associated with atrial fibrosis. Although the cause of atrial fibrosis development is not understood, its etiology is related to cardiac dysfunction, mitral regurgitation (MR), and coronary artery disease. This study focuses on determining the acute effects of myocardial infarction (MI) on left atrial (LA) function. Our hypothesis is that MI may result in changes in left ventricular relaxation, MR, and LA pressure and volume overload, leading to changes in LA geometry and mechanics, which will result in later atrial fibrosis. We studied the changes in LA size, ejection fraction, and the relative contributions of passive and active emptying, comparing controls with post-MI animals.

## Methods

Eleven Yorkshire pigs (average weight 37 ± 7 kg) were studied, including 5 control animals, and 6 pigs imaged one to two weeks after a transmural MI. The MI was induced by percutaneous balloon occlusion of left coronary artery (90 min) followed by reperfusion. All animals were imaged on a 1.5T Siemens scanner (Siemens Healthcare, Erlangen, Germany). A stack of short-axis cine images covering the left atrium were obtained with balanced SSFP, with a 1.3 x 1.3 x 3 mm^3^ spatial resolution (no gaps), and 25 frames, breath-holding and retrospective ECG-gating.

**Figure 1:**
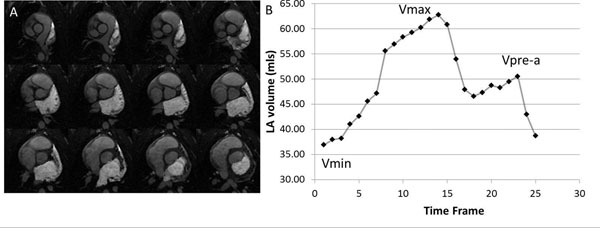
**Left atrial volume measurement.** A) Highlighted signal shows segmentation of left atrial cavity in a short-axis stack. B). Plots of LA volume vs. cardiac phase are used to determine minimum volume (Vmin), maximal volume (Vmax) and the volume prior to the atrial kick (VpreA).

All image processing was performed in Matlab (v 2014). To measure LA volume through the cardiac cycle, the cine images were segmented using thresholding, and regions of interest excluding the pulmonary veins, in each slice and each time frame. Figure 1A shows representative raw images that were analyzed with the segmentations highlighted for multiple slices. The volume at each time frame was measured (Figure 1B). The minimum LA volume (Vmin), the maximal LA volume (Vmax), the pre-atrial kick volume (VpreA) were noted (see Figure 1B). From these we calculated quantities including LA EF, LA stroke volume, all normalized to weight to account for body size.

## Results

Table 1 shows that LA Vmax, VpreA, and LA stroke volume all increased post-MI, although LA EF did not change. The percent of atrial emptying contributed by the atrial kick was not different (39 ±17% vs. 38 ±9% post-infarct).

**Table 1:** Table 1

	Controls	Post-infarct	p-value
Vmin (mls/kg)*	0.99 +/-0.16	1.2 ±0.19	0.08
Vmax (mls/kg)	1.42 ±0.31	1.89 ±0.23	0.02
VpreA (mls/kg)	1.15 ±0.20	1.47±0.18	0.02
LA EF (%)	0.30 ±0.06	0.36 ±0.07	0.15
LA stroke vol. (mls/kg)	0.44 ±0.17	0.69±0.17	0.04

*All data is indexed by weight.

## Conclusions

Using quantitative analysis of high resolution SSFP MRI we demonstrate increased LA volumes in swine post-MI. These findings are similar to those reported in patient studies of acute MI, where LA volumes are increased relative to controls (1). Future work will relate these volume changes to atrial fibrosis development.

1. QA Truong et al. Intl Journal of Cardiology, 2011.

## Funding

We gratefully acknowledge funding from NHLBI R01HL113352.

